# Temporal Associations between Daytime Physical Activity and Sleep in Children

**DOI:** 10.1371/journal.pone.0022958

**Published:** 2011-08-23

**Authors:** Anu-Katriina Pesonen, Noora M. Sjöstén, Karen A. Matthews, Kati Heinonen, Silja Martikainen, Eero Kajantie, Tuija Tammelin, Johan G. Eriksson, Timo Strandberg, Katri Räikkönen

**Affiliations:** 1 Faculty of Behavioral Sciences, Institute of Behavioral Sciences, University of Helsinki, Helsinki, Finland; 2 Finnish Institute of Occupational Health, Centre of Expertise for Work Organizations, Turku, Finland; 3 Institute of Clinical Medicine, Clinic for Children and Adolescent, Helsinki University Central Hospital and University of Helsinki, Helsinki, Finland; 4 Department of Psychiatry, University of Pittsburgh School of Medicine, University of Pittsburgh, Pittsburgh, Pennsylvania, United Stated of America; 5 National Institute of Health and Welfare, Helsinki, Finland; 6 LIKES Research Center for Sport and Health Sciences, Jyväskylä, Finland; 7 Department of General Practice and Primary Health Care, University of Helsinki, Helsinki, Finland; 8 Helsinki University Central Hospital, Unit of General Practice, Helsinki, Finland; 9 Folkhälsan Research Centre, Helsinki, Finland; 10 Vasa Central Hospital, Vasa, Finland; 11 Department of Health Sciences/Geriatrics, Unit of General Practice, University of Oulu, Oulu, Finland; University of Pennsylvania School of Medicine, United States of America

## Abstract

**Objectives:**

We examined temporal associations between objectively-measured physical activity (PA) during the day and in the evening, and sleep quantity and quality.

**Study Design:**

PA and sleep were measured by actigraphs for an average of one week in an epidemiological cohort study of 275 eight-year-old children.

**Results:**

For each one standard deviation (SD) unit of increased PA during the day, sleep duration was decreased by 0.30, sleep efficiency by 0.16, and sleep fragmentation increased by 0.08 SD units that night. For each one SD unit increase in sleep duration and efficiency the preceding night, PA the following day decreased by 0.09 and 0.16 SD units, respectively. When we contrasted days with a high amount of moderate to vigorous activity during the day or in the evening to days with a more sedentary profile, the results were essentially similar. However, moderate to vigorous PA in the evening shortened sleep latency.

**Conclusions:**

The relationship between a higher level of PA and poorer sleep is bidirectional. These within-person findings challenge epidemiological findings showing that more active people report better sleep. Since only a few studies using objective measurements of both PA and sleep have been conducted in children, further studies are needed to confirm/refute these results.

## Introduction

Short duration and poor quality of sleep are common in children [1]. Regular participation in physical activity (PA) during the day is recommended to improve sleep quantity and quality [2]. Relative to the mounting epidemiological [3], [4], [5], [6], [7], [8] and experimental [9], [10] studies in adults showing the benefits of PA on sleep, only a few epidemiological studies have addressed the associations of PA and sleep in children, with conflicting results.

In a large-scale epidemiological study comprising 68,288 US children aged 6 to 17 years, parent-reported inadequate sleep, measured by one question, was associated with over 50% higher odds of self-reported physical inactivity [11]. In another study involving children aged 12 to 18 years (N = 1,365), a self-reported low PA level was associated with an more than threefold increase in the risk of reporting insomnia symptoms [12]. In two studies with objective measurements of both sleep and PA over one 24-hour period [13], [14] (N = 519), higher levels of PA were associated with shorter sleep latency [14], but no associations were found between mean PA and sleep duration [13]. However, it should be noted that a single 24-hour period of actigraph measurement does not yield reliable estimates of sleep [15]. An experimental study in eleven 12-year-old children showed that exhaustive endurance exercise increased the amount of slow wave sleep (SWS), but did not affect sleep duration [16].

The present study examined whether PA and sleep quantity and quality, measured with wrist actigraphs worn on average for seven consecutive days and nights, were related in children. Our study design allowed us to address the temporal relationships of these associations, that is, to clarify whether PA and sleep are reciprocally related. Given the experimental evidence based on adults and on one study in children, we hypothesized that higher PA during the day would lead to better sleep quantity and quality that night in children. However, because of the study design, we also tested whether sleep quantity and quality during the night are related to PA the next day, perhaps because fatigue due to poor sleep leads to less interest in and energy for PA.

## Materials and Methods

### Participants

The children were recruited from a population-based urban cohort comprising 1,049 infants born in 1998 in Helsinki, Finland [17]. In 2006, families living close to or in the greater Helsinki area were invited to a follow-up [18], [19]. Of the 413 invited children, 321 (77.7%) agreed to participate. Non-participation was not related to a child's gender, weight, length, or head circumference at birth, birth order, mode of delivery, mother's occupational status, age, body mass index (BMI), or alcohol consumption, maternal licorice consumption, or stress during pregnancy (P-values>0.10). Non-participation was only related to more frequent self-reported maternal smoking during pregnancy (P = 0.02). Valid actigraph recordings on both PA and sleep were obtained from 297 children (50.7% girls). Three children were excluded due to a parent-reported diagnosis of a developmental delay, one due to epilepsy and two due to diabetes (N = 6 for excluded). The study was approved by the Ethics Committees of the City of Helsinki's Health Department and the Helsinki University Hospital of Children and Adolescents. In addition, each child and his/her parents gave a written informed consent.

### Measures

Sleep and daytime PA patterns were objectively measured by actigraphs (Actiwatch AW4; Cambridge Neurotechology Ltd, Cambridge, UK), which is a small accelerometer, worn on the non-dominant wrist for an average of 7 consecutive days. The children followed their usual daily schedules pertaining to place of bed and awakening times. The actigraph yields data on sleep duration (i.e., hours from sleep onset to awakening), sleep efficiency percent ((actual sleep time in hours/total time in bed)×100), sleep fragmentation percent (the addition of percentage of minutes moving and percentage of very short immobility phases [<1 minute] during the sleep/2), sleep latency (time in minutes from bedtime to sleep start), and daytime PA (counts/minute [CPM]). The data were scored with Actiwatch Activity and Sleep analyses software (version 5) with a medium sensitivity and one minute epoch duration.

To assess the intensity of PA, we used a prediction equation by Heil et al. [20] for Actical (Mini Mitter Co., Inc, Bend, OR), a newer version of Actiwatch from the same manufacturer, to derive the cut-point for moderate to vigorous physical activity (MVPA):

*Activity energy expenditure (kcal×kg^−1^×min^−1^) = 0.02299+(1.902E-5)×counts per min.*

Using this equation a cut-point of 1420 counts/min represents the 3METs (metabolic equivalent) or 0.05 kcal×kg^−1^×min^−1^ threshold for MVPA. In children, 3 METs represents, for example, the intensity of walking at a speed of approximately 4 km/h [20]. In analyses testing if the time accumulated in MVPA during waking hours is associated with sleep, we compared children with ≥60 minutes of MVPA to children that were less active. Sixty minutes of MVPA is a commonly recommended amount of daily activity for children [21]. In the absence of ready definitions for a high activity count in the evening time, we used 30 minutes as a cut-off for MVPA between 6 p.m. and 9 p.m.

### Sleep

The children were instructed to press an event marker on the actigraph at bed- and waking times, and the parents were instructed to keep a sleep log on bed- and waking times, temporary pauses in actigraph registration (e.g., while taking a shower), and on events that might affect sleep or physical activity (e.g. illness, pain, injury or travel). The activity data were visually inspected to detect significant discrepancies between the sleep log, event markers, and the activity pattern. If there were several event markers for one night, the most recently entered was used and compared with the sleep log. If the sleep log was not with the event marker within 10 minutes of the event marker, the event marker was used to define the bedtime. We found high compliance between the sleep log registrations and the event markers; for 71% of the participants, no discrepancies were found; for 21%, a discrepancy was found for one or two nights; and for 8%, a discrepancy was found for three or more nights. As previously reported, only 66 (3%) nights out of a total of 2,206 recorded nights were excluded from further analyses because parents reported a change in normal life, for example, illness or travel [22].

### Daytime physical activity

The analysis window for daytime was defined as the time the child was awake between 9 a.m. and 9 p.m. Mean PA CPM was calculated daily, such that only days with data available for at least 10 hours out of the possible 12 hours were included in the analyses, which led to the exclusion of 87 (5%) days out of a total of 1,734 recorded days. In addition, all periods during the day with no detected movement during ten consecutive epochs (i.e., 10 minutes) were recorded as missing values (were considered as activities such as showering, etc.). Also, we matched sleep and PA data in such a way that no PA was recorded (even when slight movements were detected) if the child was asleep according to the sleep actigraph data. For further analyses, we divided the day into two periods, between 9 a.m. to 6 p.m. (day) and from 6.01 p.m. to 9 p.m. (evening).

### Data reduction

To study whether PA the preceding day was related with sleep quantity and quality the following night, and whether preceding sleep quantity and quality was related with PA the following day, we formed two sets of data according to the date of recording. The first set of data linked PA i the preceding day with sleep measures the following night, and resulted in an average of 5.6 (SD = 1.4, range 3–9) day-night pairs (N for children = 275). These data allowed us to address the temporal relations of PA on sleep. The second set of data linked sleep the preceding night with PA the following day, and resulted in an average of 5.5 (SD = 1.5, range 3–9) night-day pairs (N for children = 269). These data allowed us to address the temporal relations of sleep on PA. Those children who had two or fewer day-night (n = 10) and night-day (n = 17) pairs were excluded.

### Covariates

The covariates included the child's gender, age at testing, body mass index (BMI; kg/m^2^) calculated from weight and height measured by the research nurse during a clinical visit, highest parental self-reported level of education, parent-report of any physician-diagnosed chronic disease in the child, and the season (summer from May to August vs. other).

### Statistical Analyses

Multi-level hierarchical regression analysis with a maximum likelihood estimation method and AR(1) error covariance structure was used as the primary data analytical tool to examine the temporal, within-person relations between PA and sleep. In analyses to test whether PA during waking hours was associated with sleep duration, latency, fragmentation, and efficiency that night, PA represented the within-person independent variables and sleep parameters the within-person dependent variables. In analyses to test whether sleep duration, latency, and efficacy during the night were associated with PA the following day, sleep parameters represented the within-person independent variables and PA the within-person dependent variables. Sleep duration, latency, and efficiency as independent and dependent within-person variables were tested in separate models. To facilitate interpretation of the bi-directional associations between PA and the sleep parameters, we transformed all sleep and activity variables to gender-specific z-scores. This allowed us to compare the effect sizes of the temporal associations in standard deviation [SD] units. Finally, we analyzed with the mixed models whether the time spent in MVPA during waking hours, and specifically in the evening time were associated with sleep that night. The child's gender, age at testing, BMI, the season at the time of assessment, and parental level of education represented the between-person covariates. We used SPSS version 17 software in all the analyses.

## Results

### Initial analyses

Table 1 shows the characteristics of the study sample by gender. Over half of the children came from families with a high level of parental education. As reported previously [23], boys slept significantly less and spent less time in bed compared to girls. In addition, boys were physically more active during daytime and spent more time in moderate to vigorous PA across the whole study period compared to girls.

**Table 1:** Characteristics of the participants (N = 275).

	Girls N = 142		Boys N = 133		P
Variable	Mean/N	SD/%	Mean/N	SD/%	
Age (years)	8.1	0.3	8.2	0.3	0.05
Weight (kg)	28.3	5.3	29.5	7.7	0.12
Height (cm)	130.4	5.3	132.2	5.8	0.01
Body Mass Index (kg/m^2^)	16.6	2.1	16.8	4.2	0.03
**Daytime physical activity**					
Daytime physical activity (mean counts/min)	587.8	255.7	655.7	245.4	0.03
Minutes spent in moderate to vigorous activity before from 9 a.m. to 6 p.m.	52.2	48.2	69.2	54.1	0.001
Minutes spent in moderate to vigorous activity from 6.01 p.m to 9 p.m.	15.7	18.9	18.7	21.8	0.004
**Sleep**					
Time in bed	9:42	0:24	9:32	0:26	0.001
Sleep duration (h:min)	8:30	0:39	8:16	0:39	0.003
Sleep latency (h)	0:18	0:10	0:20	0:12	0.18
Sleep fragmentation (%)	15.5	6.3	16.4	4.6	0.20
Sleep efficiency (%)	84.6	6.1	83.3	5.8	0.07
**Level of parental education** (highest in the family)					
High school diploma	16	11.3	22	16.5	0.29
Vocational education	44	31.0	29	21.8	
Bachelor's degree	25	17.6	24	18.0	
Master's degree or equivalent or higher	57	40.1	58	43.6	
**Parent-reported history of chronic disease**					
Allergic rhinitis	11	7.7	11	8.3	0.55
Other allergy	20	14.1	25	18.8	0.18
Asthma	6	4.2	11	8.3	0.13
Atopic eczema	13	9.2	19	14.3	0.15
Minor developmental defect	6	4.2	6	4.5	0.59
Dysphasia	3	2.1	5	3.8	0.34
Dyslexia	4	3.3	1	0.8	0.20

### Is PA during the day related with sleep the following night?

For each SD unit of PA in the waking hours, sleep duration decreased by 0.30 SD (95 percent confidence interval [95% CI] −0.36 to −0.25) units, sleep efficiency decreased by 0.16 SD (95% CI −0.20 to −0.11) units, and sleep fragmentation increased by 0.08 SD (0.02 to 0.13) units that night (Table 2).

**Table 2:** Results of mixed model analyses showing within-subject associations between physical activity in the preceding day and sleep quantity and quality during the following night.

	Sleep							
	Duration		Efficiency		Fragmentation index		Latency in minutes	
	SD unit change (95% CI)	P	SD unit change (95% CI)	P	SD unit change (95% CI)	P	SD unit change (95% CI)	P
Physical activity (mean counts over the entire day)*	−0.30 (−0.36 to −0.25)	<0.001	−0.16 (−0.20 to −0.11)	<0.001	0.08 ( 0.02 to 0.13)	0.007	0.00 (−0.06 to 0.07)	0.94
Moderate to vigorous activity from 9 a.m. to 6 p.m. (more than 1 hour vs. less)	−0.21 (−0.31 to −0.12)	<0.001	−0.12 (−0.19 to −0.05)	0.001	0.07 (−0.01 to 0.15)	0.10	0.03 (−0.07 to 0.13)	0.59
Moderate to vigorous activity from 6.01 p.m. to 9 p.m. (more than 30 min vs. less)	−0.16 (−0.27 to −0.05)	0.004	−.04 (−0.12 to 0.04)	0.30	0.00 (−0.08 to 0.09)	0.97	−0.12 (−0.24 to −0.00)	0.04

All models are adjusted for sex, current BMI, age, socioeconomic status of the family, season, and history of any chronic disease.

*SD unit change refers to change in the dependent variable according to an increase of one standard deviation unit in the mean daytime physical activity.

95% CI refers to 95 percent Confidence Interval.

**Figure 1:**
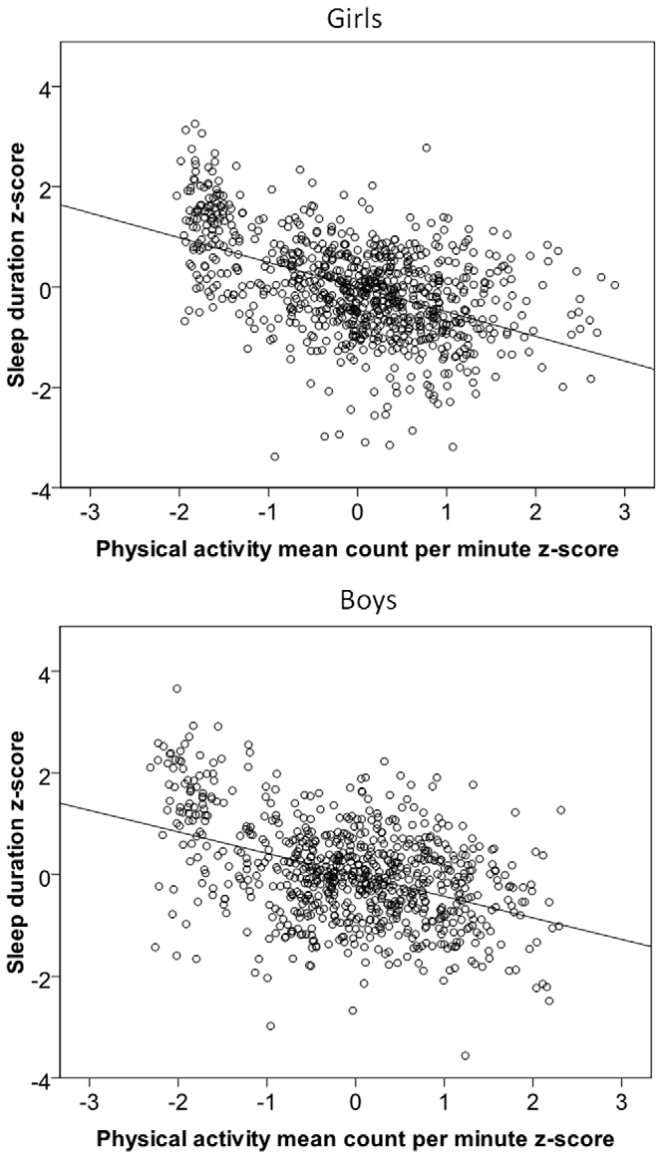
The associations between the physical activity in the preceding day and sleep duration in the following night in girls and boys.

Child's gender moderated the associations between PA and sleep duration (P for interaction <0.03; p>0.07 for other interactions), with stronger effects among girls than boys. However, higher PA was significantly associated with decreased sleep duration in both genders (girls: −0.38, 95% CI −0.46 to −0.30, P<0.001; boys; −0.21, 95% CI −0.30 to −0.14, P<0.001). Figure 1 shows the relations between PA and sleep separately for girls and boys.

To examine whether these associations were driven by the PA during the evening time in particular, we tested the associations between PA from 9 a.m. to 6 p.m. (Day PA), and from 6.01 p.m. to 9 p.m. (Evening PA). Day and Evening PA were highly correlated (r = 0.66, P<0.001). The associations both between Day PA and Evening PA and sleep the following night were similar to the associations using PA across the entire day as a predictor (P-values for all associations <0.001), except for one: PA after 6 p.m. was not associated with sleep fragmentation during the following night (P>0.08).

Table 2 shows the results from the analyses to test whether moderate to vigorous intensity PA was associated with sleep the following night. Days with MVPA for more than one hour was associated with 0.21 SD (95% CI −0.31 to −0.12) units shorter sleep and 0.12 SD (95% CI −0.19 to −0.05) units lower sleep efficiency that night, compared to days with less than one hour of MVPA. If there was more than 30 minutes of MVPA in the evening after 6 p.m., the duration of sleep during that night was 0.16 SD (95% CI −0.27 to −0.05) units shorter, and the sleep latency shortened by 0.12 SD (95% CI −0.24 to −0.00) units.

### Is sleep during the night related with PA the next day?

For each SD unit increase in sleep duration and efficiency the preceding night, PA the following day decreased by 0.09 SD (95% CI −0.13 to −0.05) and 0.16 SD (95% CI −0.21 to −0.11) units, respectively (Table 3). For each SD unit increase in the fragmentation index or latency, PA increased by 0.05 SD and 0.04 (95% CIs 0.00 to 0.09; 0.0 to 0.08, respectively) units the following day. Gender did not moderate the significant associations.

**Table 3:** The results of mixed model analyses showing the within-subject associations between sleep quantity and quality in the preceding night and physical activity during the following day.

	Daytime physical activity	
	SD unit change (95% CI)	P
Sleep		
Duration	−0.09 (−0.13 to −0.05)	<0.001
Efficiency	−0.16 (−0.21 to −0.11)	<0.001
Fragmentation index	0.04 (−0.00 to 0.09)	0.07
Latency	0.03 (−0.00 to 0.07)	0.08

All models are adjusted for sex, current BMI, age, socioeconomic status of the family, season, and history of any chronic disease.

SD unit change refers to change in standard deviation unit according to an increase of one standard deviation unit in sleep quality or quantity.

95% CI refers to 95 percent Confidence Interval.

## Discussion

Our study in 8-year-old children sought to determine the within-person associations between sleep quantity and quality, and daytime PA by means of an objective actigraph measurement over an average period of one week. The length of the recording period allowed us to address the temporal direction of the associations. Our findings indicated that PA and sleep were significantly related, such that a higher level of PA during the waking hours was associated with poorer sleep that night, and poorer sleep during the night was associated with a higher level of PA the following day. These associations were not driven by PA specifically during the evening and were obtained in analyses comparing moderate/vigorous PA to less active PA days. With the exception of more moderate to vigorous PA during the evening being associated with shorter sleep latency that night, our results were opposite to our hypotheses. Our hypotheses were based on previous epidemiological studies reporting that more physically active children had better sleep as reported by parents or children themselves [10], [11]. However, self-reports have shown to correlate moderately with objective measurements, with subjective reports tending to overestimate the amount of daily PA [24], and especially sleep duration [25]. For instance, sleep duration measured by actigraph or polysomnography in 8-year-old children has been generally reported to be ≈8 hours [22], [26], [27], [28], whilst the average is 10.4 hours in self- or parent reports [29].

The association between objectively measured PA and sleep among school-aged children has previously been explored in only two studies [13], [14]. Contrary to our findings, Nixon et al. [13] did not report a significant association between sleep duration and PA. With regard to sleep latency, Nixon et al.[14] showed that a higher PA level during the day was associated with shorter sleep latency. In our analyses, higher PA was not significantly associated with sleep latency the same evening, but 30 minutes or more spent in moderate to vigorous PA during evening was followed by shorter sleep latency.

Epidemiological studies among adults and (pre-)adolescents have produced a mounting literature showing a J-shaped curve between sleep duration and BMI and risk for type 2 diabetes [30], [31], [32], [33]. Our findings may then shed light on the pathways that may account for the elevated risk associated with long sleep duration.

Results associating increased PA with poorer sleep, and *vice versa*, may reflect an underlying factor common to both. One such factor could be the inherent activity level of child, which seems to manifest itself in a higher activity level during both the day and the night. This is an intriguing observation, given that activity level as a temperament characteristic is among the traits which show most continuity over childhood [34], [35], and show a relatively high heritability estimate (36%) [36]. From this perspective we suggest that the “trait” activity level may not only explain PA during the day, but may also define the level of activity during the night, which by definition shortens the actual sleep time. Other mechanisms may also be involved. Several theories, such as those based on thermoregulatory, body restoration, and energy conservation hypotheses, have been suggested to explain the positive effects of PA on sleep in adults [9]. An exciting possibility relates to the function of the hypothalamic-pituitary-adrenal axis. We have recently demonstrated that poor sleep in children is associated with higher cortisol levels upon awakening, at bedtime, and in response to psychosocial stress [37] Vigorous PA has, in turn, been associated with increases of serum and salivary cortisol levels [38], [39].

The main strengths of this study relate to the large community-based sample and the objectively measured data on PA and sleep over multiple consecutive days and nights. Actigraphs are widely used in epidemiological research to measure sleep in an ecologically valid setting for the child [40]. However, actigraphy also has its limitations, such as its inability to assess static exercise and certain types of dynamic activities, for example, cycling and water sports (the devices are not water-resistant) [41], [42]. There is also speculation as to whether actigraphs placed on the wrist measure daytime PA as well as actigraphs worn around waist. It has been suggested that wrist-worn devices may be better tolerated by participants than devices placed at other locations [43], yielding then higher compliance because the device is not obstructing the natural way of living. On the walking level, there are no clear differences in PA estimates whether accelerometers are placed at hip, at wrist (as in the present study), or whether they are uni- or multiaxial [41]. While wristworn accelerometers may produce higher scores in sedentary activities compared to those obtained with a waist-worn device [44], we placed the actigraphs in the non-dominant wrist in order to eliminate activities such as writing from the physical activity counts. In addition, wrist-worn devices, from the same manufacturer as in our study, have accurately predicted energy expenditure measured by indirect calorimetry in short-term protocols in children and adults [20], [45].

Finally, it should be emphasized that temporal associations are not equal to causal relationships. It is always possible that the associations found in our study are dependent on a third factor that could not be specified here. It should also be noted that the families in the present study represent higher education background more often than in the general population, restricting the external validity of the study.

### Conclusion

Our results showed that a higher level of PA during the waking hours was related with shorter sleep duration, lower sleep efficiency, and higher fragmentation of sleep that night in school-aged children. In addition, poorer sleep at night was related to higher PA the following day. These results contradicted the mainstream of epidemiological findings showing the benefits, albeit modest, of PA on sleep. However, in line with our hypotheses, we found that a high level of moderate to vigorous PA during the evening was associated with shorter sleep latency that night. Since only a few studies using objective measurements of both sleep and PA have been conducted on children, it is clear that further studies in children are needed to confirm our results.
